# Accidental Removal of a Carotid Endovascular Stent during Oropharyngeal Mass Biopsy

**DOI:** 10.1155/2009/378683

**Published:** 2010-01-04

**Authors:** Charbel Rameh, Arnaud Deveze, Jean-Pierre Lavieille, Jacques Magnan, Melanie Sanjuan

**Affiliations:** Department of Otolaryngology Head and Neck Surgery, Nord University Hospital, Chemin des Bourrely, Marseille Cedex 13015, France

## Abstract

A 54-year-old male patient, with a history of a right mandibular adenocarcinoma, previously excised, and treated with post operative chemo- and radio-therapy, presented with a right oropharyngeal necrotic mass of several months duration. His history is pertinent for a right internal carotid endovascular stenting 2 years prior to presentation. During biopsy of his oropharyngeal lesion, a specimen of tissue was retrieved, with the carotid stent within. There was no bleeding. To the best of our knowledge, there is no such case reported in the literature. We present this case as a reminder on the importance and risks of radiation-induced necrosis and its distortion of the surrounding anatomy, especially in the presence of foreign bodies or protheses.

## 1. Introduction

Injuries to the internal carotid artery are not common. They most often result from accidental neck injuries, or from intraoperative insults in the context of oropharyngeal surgery, mostly tonsillectomy [1]. There is no case reported whereby an internal carotid artery endovascular stent was removed accidentally during a lateral pharyngeal mass biopsy, and without bleeding. Hereby we present such a case, as a reminder to be kept in mind of the proximity of the great vessels to the lateral pharyngeal wall, especially in the setting of an irradiated neck with a foreign material prosthesis or stent.

## 2. Case Report

The patient is a 54-year-old man who first presented to our department in 1988 with an adenocarcinoma of the right mandible. He underwent a right hemimandibulectomy, neck dissection, and reconstruction using iliac bone and pectoralis major flap, with postoperative chemotherapy and 70Gy radiotherapy. He did well until 2006 when he was found to have a right internal carotid artery near total occlusion, most probably post radiation in origin, with a 40% contralateral stenosis. He underwent consequently a right carotid artery angiography and endovascular stenting, and was started on anticoagulation.

**Figure 1:**
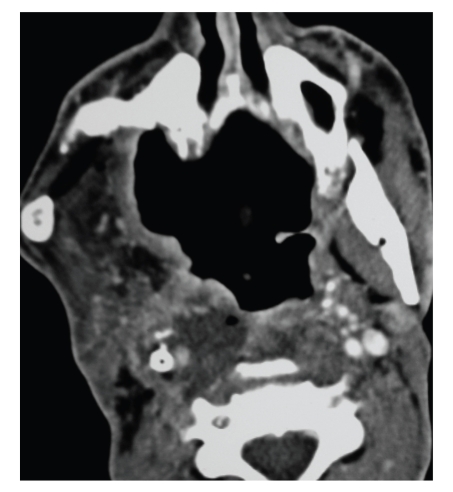
Contrast Enhanced computed tomography scan of the neck showing the right carotid stent occluded with no blood flow in the lumen. The carotid is surrounded by the ulcerative mass.

**Figure 2:**
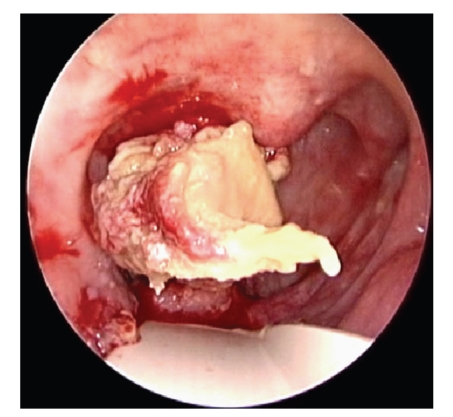
Endoscopic view of the right lateral oropharyngeal lesion.

**Figure 3:**
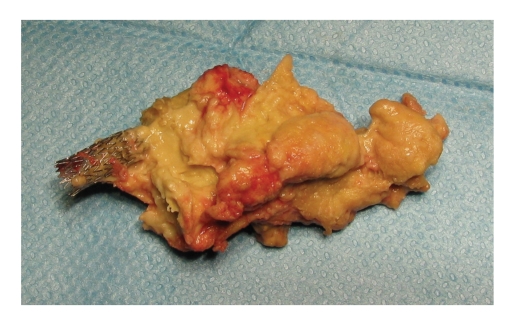
Biopsy specimen, with the carotid stent seen invaded and occluded by the mass.

On his last follow up in our clinic, he was found to have a nonhealing 5 × 3 cm necrotic mass occupying the right oropharyngeal space. Biopsies were taken from the ulcer and were inconclusive, favoring necrotic tissue. Attempts of hyperbaric oxygen therapy were unsuccessful. Therefore, he was scheduled for debridement of the ulcer and biopsy of the mass in the operating room to rule out the possibility of tumor recurrence. Preoperative Computed Tomography scanning (CT) showed the irregular ulcer at the right oropharyngeal space, with the right carotid artery stent completely occluded with no distal perfusion (Figure 1), and it was encased in the mass of necrotic tissue. In the operating room, under general anesthesia, the oropharyngeal lesion was inspected endoscopically transorally (Figure 2). Using a punch biopsy forceps, a specimen was taken from the ulcer for pathological examination. However, the mass was so rubbery and consistent that a large piece was excised, with a net-shaped cribriform metallic object encased within. On inspection, the carotid endovascular stent was identified, invaded, and surrounded by the mass of necrosis that was filling the lateral oropharyngeal wall (Figure 3). The patient had no bleeding. The vascular surgery team was consulted into the operating room, to assess the possibility of any secondary bleeding. They recommended close surveillance of the patient for 72 hours since there was no evidence of bleeding and since the ICA was already completely obstructed, with practically a very minimal risk of vessel rupture. The patient was discharged home a few days later in a good condition. Pathology revealed necrotic tissue with absence of tumor cells.

## 3. Discussion

Injuries to the internal carotid artery (ICA) are uncommon. Most cases have been described in the context of head trauma or oropharyngeal surgery, mainly in relation to tonsillar surgery [1, 2]. In our report, we go beyond tonsillectomy to describe the first case of a carotid endovascular stent removal during a lateral oropharyngeal mass biopsy. We have performed a Medline literature review dating back to 1950, and to the best of our knowledge, there is no similar reported case.

Physicians, in particular otolaryngology residents in training, have always been warned about the potential risk of digging deep in the tonsillar fossa while doing an extracapsular tonsillectomy, and advised to “stick to the tonsillar capsule” for more safety. The reason is that the ICAs are relatively superficially located posterolateral to the lateral pharyngeal wall or tonsillar fossa. In fact, there are multiple studies describing the relative location of the ICA with respect to the tonsillar fossa. Hendrix et al found that the ICA was located anterior to the posterior pillar by a distance that is grossly equivalent to 40% the width of the tonsillar fossa [3]. Deutsch et al. described this distance to be roughly around 25 mm in the adult population, but it can occasionally be much smaller [4]. This means that any incidental movement with a sharp instrument during oropharyngeal interventions might potentially injure the ICA, and cause severe hemorrhage. If we add to this the incidence of carotid course anomalies such as kinking, tortuosity, or coiling (4–66%) [5], we can see that a seemingly simple surgery might sometimes unwillingly turn disastrous. After performing so many uneventful simple tonsillectomies, our meticulous sharp attention as surgeons might sometimes be blunted, and a tiny pulsating mass in the lateral pharyngeal wall might escape our detection.

Another point of importance is the effect of radiation therapy on the ICA. Though our patient received his neck radiation therapy 20 years ago, delayed effects of radionecrosis have been described up to 50 years later [6]. Radiation necrosis, with the high dose of 70Gy that was administered to our patient, is probably the cause of the ulcer's poor healing capacity. Radiated vascular endothelia are damaged, with secondary obliteration and fibrosis of the vessels [6]. Some authors have even suggested that radiation arteritis might be a contraindication to stenting, because these vessels often restenose [7], such as in our patient whose right ICA was stented only two years ago. Carmody et al. described a 22% prevalence of more than 70% stenosis of the carotid artery lumen in radiated necks compared to 4% in controls [8].

On preoperative contrast enhanced CT scans done 1 month before our biopsy, our patient's right ICA had a normal course with no apparent tortuosity, coiling, or kinking. However, he had a total occlusion of his stent with no flow into the distal carotid on the right side, the contralateral circulation probably taking over the blood supply of the occluded side. The biopsy was done in the operating room under general anesthesia, in anticipation of any complications that might occur, considering the patient's complex medical background. The mass of necrosis had apparently engulfed the ICA and invaded it, contributing to the obstruction of the endovascular carotid stent and of the ICA. The previous radiation therapy that the neck was exposed to had probably played its role in thrombosing the stent, causing a distal carotid wall necrosis, following which the stent had become denuded and adherent to the bulk of the invading oropharyngeal mass.

Our patient was lucky that the carotid was already totally occluded, as could be seen preoperatively on the scan, and that stent removal happened uneventfully without any bleeding. In retrospect, it would have been better to use a scalpel to sharply biopsy the lesion, avoiding the traction made by a punch biopsy forceps. This case ended smoothly, but it could have possibly turned catastrophically. This raises a red flag. We should be careful and always remember the proximity of the great vessels when working laterally in the pharynx, such as while performing a tonsillectomy or tonsillar biopsy, especially in radiated necks. Computed tomography and magnetic resonance imaging are key, giving us spatial information on the surrounding structures. It is of great importance to recognize the position of the ICA prior to performing the surgery, especially in cases where anatomy is distorted by a neoplastic or necrotic invasive process.

There is no real consensus as to the management of an intraoperative ICA rupture. It is mainly because this is a very rare occurrence, which is often lethal short of aggressive action to stop the bleeding. In this paper, it is not our aim to discuss the treatment of such scenarios.

## 4. Conclusion

This case highlights the impact of radiation-induced necrosis and draws our attention to the importance of close follow up of endovascular stents or other cervical instrumentation in the setting of an irradiated neck. The increased risks of surgical interventions in adjacent areas are to be kept in mind, and especially during manipulation of the pharyngeal wall, being a boundary to some underlying vital structures such as the ICA.
